# Molecular Epidemiology of Enteroaggregative *Escherichia coli* (EAEC) Isolates of Hospitalized Children from Bolivia Reveal High Heterogeneity and Multidrug-Resistance

**DOI:** 10.3390/ijms21249543

**Published:** 2020-12-15

**Authors:** Enrique Joffré, Volga Iñiguez Rojas

**Affiliations:** 1 Centre for Translational Microbiome Research (CTMR), Department of Microbiology, Tumor and Cell Biology (MTC), Karolinska Institutet, 171 77 Stockholm, Sweden; 2 Instituto de Biología Molecular y Biotecnología (IBMB), Carrera de Biología, Universidad Mayor de San Andrés (UMSA), La Paz 10077, Bolivia; volgavir@yahoo.com

**Keywords:** enteroaggregative *E. coli*, infant diarrhea, genetic diversity, severity, multidrug-resistant *E. coli*, Bolivia

## Abstract

Enteroaggregative *Escherichia coli* (EAEC) is an emerging pathogen frequently associated with acute diarrhea in children and travelers to endemic regions. EAEC was found the most prevalent bacterial diarrheal pathogen from hospitalized Bolivian children less than five years of age with acute diarrhea from 2007 to 2010. Here, we further characterized the epidemiology of EAEC infection, virulence genes, and antimicrobial susceptibility of EAEC isolated from 414 diarrheal and 74 non-diarrheal cases. EAEC isolates were collected and subjected to a PCR-based virulence gene screening of seven virulence genes and a phenotypic resistance test to nine different antimicrobials. Our results showed that atypical EAEC (a-EAEC, AggR-negative) was significantly associated with diarrhea (OR, 1.62, 95% CI, 1.25 to 2.09, *p* < 0.001) in contrast to typical EAEC (t-EAEC, AggR-positive). EAEC infection was most prevalent among children between 7–12 months of age. The number of cases exhibited a biannual cycle with a major peak during the transition from warm to cold (April–June). Both typical and a-EAEC infections were graded as equally severe; however, t-EAEC harbored more virulence genes. *aap*, *irp2* and *pic* were the most prevalent genes. Surprisingly, we detected 60% and 52.6% of multidrug resistance (MDR) EAEC among diarrheal and non-diarrheal cases. Resistance to ampicillin, sulfonamides, and tetracyclines was most common, being the corresponding antibiotics, the ones that are frequently used in Bolivia. Our work is the first study that provides comprehensive information on the high heterogenicity of virulence genes in t-EAEC and a- EAEC and the large prevalence of MDR EAEC in Bolivia.

## 1. Introduction

Diarrheal disease remains a significant public health problem, and it is the third leading cause of child mortality globally. In countries from Sub-Saharan Africa, South Asia, and Latin America, diarrhea is a major cause of death among children younger than two years old [1]. Enteroaggregative *Escherichia coli* (EAEC) is an emergent bacterial pathogen implicated in endemic diarrhea in developing and developed countries and causing acute and persistent diarrhea (>14 days) in children and adults [2]. In some countries of the developing world, EAEC was one of the most prevalent agents associated with diarrhea in children less than five years of age [3,4,5,6,7]. During EAEC infection, the clinical manifestations are often characterized by watery or mucoid diarrhea with or without blood and low-grade fever. EAEC colonizes the small and large bowels and can lead to mild inflammation in the colon [8]. Long-term consequences of EAEC colonization cause growth retardation among children with or without diarrhea [9].

The gold standard for EAEC identification is the detection of the aggregative adherence (AA) pattern on HEp-2/HeLa cells. It requires specific laboratory conditions, and it is time-consuming; therefore, molecular techniques such as PCR amplification are a great advantage, particularly in developing settings for extensive epidemiological studies [10]. EAEC expresses a heterogeneous array of virulence factors encoded on the bacterial chromosome or the EAEC-specific pAA plasmid, associated with the bacteria’s ability to aggregate (AA) [11,12,13]. Each virulence gene plays a role in the disease, although the pathogenesis of EAEC infection has not been entirely resolved [14]. For EAEC identification by PCR, numerous virulence genes were used as virulence markers; however, the plasmid genes *aatA* (pCVD432) and/or *aggR* were widely used among epidemiologic studies [10,15,16]. An increasing number of studies have complemented the chromosomal gene *aaiC* [12,17,18,19,20].

The pAA plasmid harbors the transcriptional activator of the AraC/XylS class called AggR, which regulates the expression of the aggregative adherence fimbriae (AAFs: AAF/I; *aggA,* AAF/II; *aafA,* AAF/III; *agg3A*, AAF/IV; *aff4A and* AAF/V; *agg5A*) are involved in EAEC adherence to the intestinal epithelium [21,22,23]. Based on the presence of the *aggR* gene, strains of EAEC can be classified into typical (*aggR*-positive) and atypical strains (*aggR*-negative) [24]. Other plasmid genes regulated by AggR are dispersin (*aap*) [25] and the dispersin translocator Aat (*aatA*) [26]. The chromosome-encoded AAI (*aai*) type VI secretion system was also reported to be regulated by AggR [24]. The non-AggR-regulated plasmid virulence genes are the EAEC heat-stable enterotoxins 1 (EAST1; *astA*) [27] and the plasmid-encoded toxin (Pet; *pet*) [28]. Other virulent genes are located on the bacterial chromosome, such as a 116-kDa secreted mucinase (Pic; *pic*) [29] and yersiniabactin biosynthesis (*irp2*) [30]. These factors are not present among all EAEC strains and often are found in EAEC isolates from non-diarrheal cases. A later study has shown that EAEC isolates can carry additional putative virulence genes described in other pathogenic *E. coli* strains [31]. Virulence genes of EAEC vary geographically, contributing to EAEC heterogeneity and making the identification of EAEC challenging [32].

Treatment of bacterial diarrhea use as a universal method of oral rehydration (ORS) therapy [33], intravenous fluids, breastfeeding, continuous feeding, zinc tablets, and community-based management practices by parents/guardians [34]. Antibiotics treatment is usually restricted to bloody diarrhea and cholera cases with severe dehydration, but antibiotics are nevertheless frequently prescribed [35]. Thus, antimicrobial-resistant *E. coli* has become a worldwide health concern, especially in developing nations where the unregulated sale of antibiotics, patients’ expectations, rising income, and limited public health response have helped drive the emergence of resistance [36]. The rapid increase of resistance in diarrheal pathogens pose a serious threat [37].

In our previous study [7], EAEC was the most prevalent diarrheagenic *E. coli* causing 11% of all hospitalizations for acute diarrhea among children less than five years old. This report highlighted the importance of EAEC as an emerging pathogen in the region and the urge to be followed up. The collection of EAEC isolates from children with or without diarrheal disease from two main cities of Bolivia was further characterized, and to our best awareness, no studies focusing on EAEC characterization have been done yet in Bolivia. Hence, this study investigates different aspects of the EAEC infection, such as seasonality and disease severity and the prevalence of selected virulence genes and antimicrobial resistance (AMR) of the circulating EAEC isolates.

## 2. Results

### 2.1. Atypical EAEC is Associated with Diarrhea

From the 3943 stool samples collected from children with an acute diarrheal disease during the 4-year study period (2007–2010) and including two cities of Bolivia (La Paz and Cochabamba), EAEC was detected in 440 stool samples (11.2%) as a single associated diarrheagenic *E. coli* using *aatA*-specific PCR assay. In parallel, 74 EAEC isolates (7.2%) were detected from 1026 non-diarrheal stools from hospitalized children admitted for a reason other than acute diarrhea. Among EAEC isolates, we targeted the *aggR* gene as a PCR marker to identify the EAEC subtypes: typical (*aggR* positive) and atypical (*aggR* negative). Our data from Table 1 revealed that from the total cases of diarrhea, 254 (6.4%) were caused by atypical EAEC (a-EAEC), while 186 (4.7%) were typical EAEC (t-EAEC). Typical and atypical EAEC isolates were also identified in the same proportion within the non-diarrheal cases. Similar to EAEC, a-EAEC isolates were found to be associated with diarrhea (OR, 1.62, 95% CI, 1.25 to 2.09, *p* < 0.001) in contrast to t-EAEC (Table 1).

### 2.2. High Prevalence of EAEC Infections During the Cold-Dry Season and Among Younger Children

**Figure 1:**
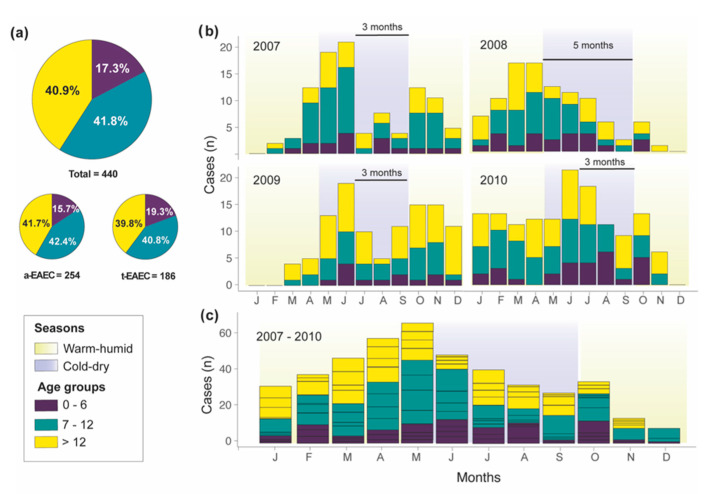
Age-wise and seasonal distribution of EAEC isolated from the stool samples of Bolivian children with acute diarrhea. (**a**) Age group stratification of EAEC isolated from diarrheal and non-diarrheal cases. (**b**) Seasonal distribution of age groups of EAEC isolates from diarrheal cases broken down per year or (**c**) accumulated.

The age distribution of the children enrolled in the study indicated that 91.1% (*n* = 401) of the diarrheal cases were among children less than two years of age. In non-diarrheal cases, the percentage was 63.5% (*n* = 47). As it is shown in Figure 1a and Table S1, EAEC was most prevalent among children between seven to twelve months of age (41.8%), followed by children older than 12 months (40.9%) and younger than seven months (17.3%). Age distribution between diarrheal cases of t-EAEC or a-EAEC was similar, except with children between 7–12 months of age that were statistically twice more likely to be infected by a-EAEC (OR 2.08, 95% CI, 1.05 to 4.26, *p* = 0.0369).

The temporal and seasonal distribution, e.g., warm-humid or cold-dry, of the diarrheal cases of EAEC in conjunction with the age groups were evaluated per year and globally. As shown in Figure 1b, EAEC-related cases had a biannual cycle with a major peak during the cold-dry season (from May to September) and a minor peak in October where the transition from cold-dry to warm-humid season occurs. The biannual cycle is evident in Figure 1c, showing the cumulative number of EAEC cases from the whole study period. EAEC’s major peak started earlier in 2007 and 2008, but in 2009 and 2010 shifted towards the coldest month of the year (June and July). The minor peak continually stroked after the end of the cold-dry season (October). On average, EAEC’s peaks had a periodicity of 3.5 months (3–5 months). We did not find any specific statistical association between the number of cases of EAEC (typical and atypical) and season (cold-dry or warm-humid season) (Table S2).

### 2.3. Typical and Atypical EAEC Isolates Caused Equally Severe Diarrhea in Children

Later, we classified the EAEC into these two groups and compared the clinical manifestations and the typical and atypical EAEC associated diarrhea severity. The typical EAEC (t-EAEC) isolates are usually considered more virulent than atypical EAEC (a-EAEC) [6,24]. Only 159 cases of EAEC diarrhea had the completed clinical data; 81 were typical and 78 atypical EAEC. As is summarized in Table 2, pediatric patients infected either with t-EAEC or a-EAEC had similar clinical characteristics and disease severity, and no statistical differences were found. However, we noticed that t-EAEC tended to cause more moderate cases (72%) than a-EAEC (67.9%).

### 2.4. Typical EAEC Isolates Harbor More Virulence Genes than Atypical EAEC

We screened for seven virulence genes among the t-EAEC and a-EAEC isolates from diarrheal and non-diarrheal cases, including plasmid-borne (*aap, astA, aggA, aafA*, and *pet*) and chromone-encoded virulence genes (*irp2* and *pic*). The results summarized in Table 3 showed that *aap*, *irp2*, and *pic* were the most prevalent among all EAEC isolates and present in more than 50% of the isolates. The genes *irp2* (*p* = 0.001) and *aafA* (*p* = 0.039) were significantly more prevalent among diarrheal EAEC isolates than non-diarrheal EAEC isolates. The rest of the virulence genes were equally identified among diarrheal and non-diarrheal isolates. The combination *aap*, *irp2*, and *pic* was the most prevalent virulence genotype in diarrheal isolates (7.3%), while *pic*-only was the most prevalent in the non-diarrheal isolates (8.1%) (Table S3). The genes *astA*, *aggA*, *aafA*, and *pet* were found to a lesser extent. Within EAEC isolates from diarrheal cases, the genes *aap* (*p* = 0.002) and *aafA* (*p* = 0.02) were significantly more common in t-EAEC and not a-EAEC. The *aap* gene was also more common in non-diarrheal t-EAEC than a-EAEC (Table 3).

**Figure 2:**
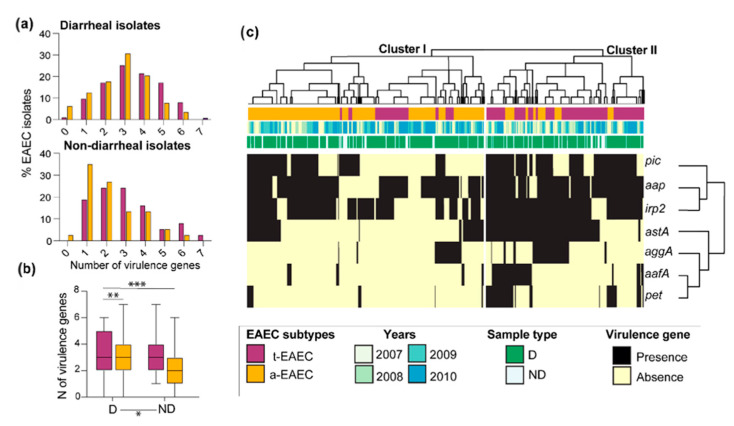
Characterization of virulence genes of EAEC isolates. (**a**) The number of virulence genes found in diarrheal and non-diarrheal EAEC isolates. (**b**) Comparison of the average of virulence genes per isolate between groups (**c**) Heatmap and hierarchal clustering of virulence gene presence and absence across EAEC isolates. Genes were clustered by similarity using Pearson coefficients. D = diarrheal case, ND = non-diarrheal case. * *p* < 0.05; ** *p* <0.01; *** *p* < 0.001.

Based on the number of virulence genes detected per strain, an average of 3.1 virulence genes/isolate in isolates from diarrheal cases was estimated, while non-diarrheal isolates harbored 2.6 (*p* < 0.05). t-EAEC and a-EAEC isolates harbored in average 3.4 genes/isolate and 2.8 genes/isolate (*p* < 0.01), respectively. The lowest number of genes per isolate was found among non-diarrheal a-EAEC isolates with 2.3 genes/isolate vs. the three genes/isolate from the non-diarrheal t-EAEC isolates (*p* < 0.001) (Figure 2a,b). Fourteen (3.2%) diarrheal and one non-diarrheal (1.4%) isolates were negative to all the virulence genes included in this study (Table S3).

In Figure 2c, we constructed a heatmap to screen the distribution of virulence genes found on t-EAEC and a-ETEC isolates along the four-year study period and whether specific profiles were associated with diarrheal cases. The clustering patterns of virulence genes did not reveal a particular clustering of diarrheal or non-diarrheal isolates, indicating that similar profiles of virulence genes circulated throughout the study period and between diarrheal and non-diarrheal cases. Two clusters were identified, of which cluster II enclosed most of the t-EAEC isolates characterized by having a more extensive number of virulence genes, as we showed previously. Cluster I, on the other hand, included the majority of a-EAEC. The genes *pic*, *aap,* and *irp2* were more likely to be found together than the rest of the virulence genes, and both t-EAEC and a-EAEC isolates shared them. The *astA*, *aggA*, *aafA*, and *pet* were more restricted to t-EAEC isolates, which harbors the master regulator *aggR* and regulates fimbrial genes (*aggA* and *aafA)*. These data suggest that the composition between typical and atypical isolates is not entirely different, and they share many virulence genes. Still, there is a specific combination of virulence genes that branch them apart.

### 2.5. High Prevalence of MDR in EAEC Isolated from Diarrheal and Non-Diarrheal Cases

**Figure 3:**
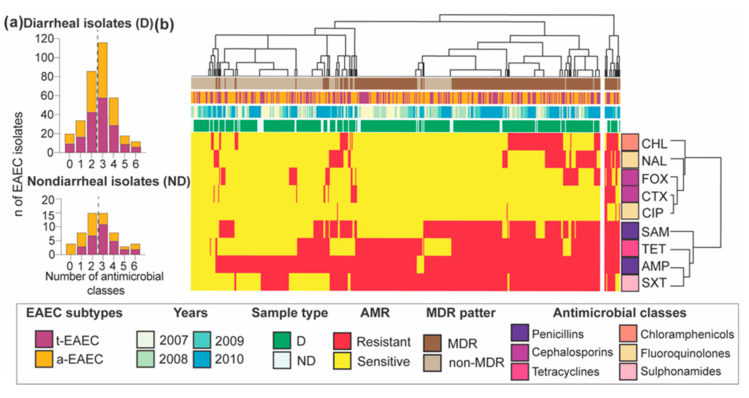
Prevalence of AMR phenotypes from t-EAEC and a-EAEC isolated from diarrheal and non-diarrheal cases. (**a**) Histograms illustrating the number of drug classes to which t-EAEC and a-EAEC isolates were phenotypically resistant. (**b**) The percentage of AMR phenotypes stratified by year. D = diarrheal; ND = non-diarrheal; AMP, ampicillin; SAM, ampicillin-sulbactam; SXT, trimethoprim-sulfamethoxazole; CHL, chloramphenicol; FOX, cefoxitin; CIP, ciprofloxacin; NAL, nalidixic acid; TET, tetracycline; CTX, cefotaxime; MDR, multidrug-resistant. D = diarrheal case, ND = non-diarrheal case.

Susceptibility testing of the diarrheal and non-diarrheal EAEC isolates to 9 different antibiotics revealed resistance to all of the antimicrobials tested with multidrug-resistance (MDR) in more than half of the isolates. For example, 60% of diarrheal and 52.6% of non-diarrheal isolates were multidrug-resistant, meaning they were resistant to at least three classes of antimicrobials, and 2.7% and 7% were resistant to the six antibiotics classes tested; however, no statistical differences were found between these two groups (Table 4 and Figure 3a). Resistance to cephalosporins such as cefoxitin and cefotaxime were significantly lower among diarrheal isolates than non-diarrheal isolates. In contrast, significantly more diarrheal isolates were resistant to the older antimicrobial trimethoprim-sulfamethoxazole than non-diarrheal isolates (*p* = 0.0024). Between t-EAEC than a-EAE, the first had twice more resistant isolates to cefotaxime than the atypical subtype (*p* < 0.05). Overall, the β-lactam antibiotic ampicillin reported the highest number of resistant isolates, followed by sulfonamides and tetracyclines. Resistance to fluoroquinolones, especially ciprofloxacin, which is considered widely used to treat diarrhea in Bolivia, was the lowest (Table 4). The heatmap of the temporal distribution of AMR profiles in Figure 3 showed co-occurrence of resistant phenotypes of penicillins such as ampicillin (AMP) or ampicillin-sulbactam (SAM), and tetracycline (TET) or sulphonamides (SXT), which could be due to the acquisition of AMR genes co-transferred via mobile elements. MDR isolates did not show any particular clustering pattern regarding the year of isolation, diarrheal or non-diarrheal cases, or EAEC subtype type.

## 3. Discussion

This study represents the first comprehensive report of the characterization of diarrhea caused by EAEC (typical or atypical). The phenotypic and genotypic heterogeneity of the bacterial isolates recovered from acute diarrheal and non-diarrheal cases from Bolivian children during four-year surveillance of diarrheagenic bacteria in two Bolivian cities. In our previous study [7], EAEC was found as the most prevalent diarrheagenic *E. coli* associated with acute diarrhea in Bolivian children, and the study of this pathovar is highly relevant to the region. Like studies in industrialized countries where EAEC is not a serious health problem but causes sporadic outbreaks and costly hospital admission, the prevalence ranges from 2% to 12% [38,39]. However, in Latin American countries such as Perú [40,41], Brazil [18,42,43,44], and Paraguay [45], studies have pointed out EAEC as the most isolated bacterial pathogen from children with acute diarrhea with a prevalence that ranges from 14% to 45%. Other studies associate EAEC with persistent diarrhea, sporadic outbreaks in industrialized nations, and adult travelers to endemic regions suggest that this emergent pathogen is highly heterogeneous [45].

Several similar studies indicate a significant heterogeneity of virulence markers among EAEC isolates, and its detection can be challenging. Here we used the *aatA* gene, which corresponds to the former EAEC probe CVD432, to identify EAEC. Further identification of the *aggR* gene’s presence allowed us to classify the EAEC isolates into typical and atypical EAEC, similar to other studies [6,14,46,47]. The prevalence of the a-EAEC was surprisingly higher and significantly associated with diarrhea than t-EAEC and non-diarrheal EAEC isolates. The majority of the epidemiological studies have reported t-EAEC isolates as the predominant subtype of EAEC isolated associated with the disease [6,32,46,48], while other studies have not been able to associate t-EAEC (AggR-positive) with diarrhea [19,49,50]. Therefore, the association of *aggR*-positive strains and diarrhea is not consistent.

EAEC infection was more prevalent among children with diarrhea less than two years of age, with two epidemiological peaks during the seasonal transition. The first EAEC peak of April, May, and June, overlapped with the maximum number of hospitalizations related to diarrhea, being a large proportion of them due to rotavirus, as we previously reported [7]. Additionally, we observed that other bacterial pathogens such as ETEC had a peak in May and a dramatic reduction of cases towards August. Along the Bolivian Andes, this period is characterized by an important reduction of temperature and rainfall, where the coldest nights are often reported. Our observation of a large peak in the transition to winter contrasts the notion that infections caused by bacterial pathogens are mostly associated with warmer and humid months (or summer), where conditions such as high temperature, rain, and humidity favor bacterial proliferation and propagation [51]. For example, the GEMS study on pathogens with a strong association with diarrhea, such as ETEC secreting ST associated the warm and rainy weather in Asia (Pakistan, India, and Bangladesh) and an African country (Mozambique) with an increased number of diarrheal cases [52]. A four-year follow-up of EAEC prevalence allowed us to observe biannual periodicity, with approximately 4–5 months gap between the peaks suggesting that seasonal transition might play an indirect role in EAEC prevalence that could facilitate the transmission of the pathogen into the population. Additional socioeconomic factors such as crowded settings or households with poor hygiene, sanitation, and contaminated water could exacerbate the propagation of EAEC during the cold-dry season. Studies with more recent EAEC prevalence data need to be considered to evaluate whether the biannual periodicity of EAEC remains univariable even after the severe consequences of climate change and water management that La Paz and Cochabamba cities experienced in the last decade [53].

The severity of the diarrheal illness in children by EAEC was evaluated and compared between typical and atypical subtypes with no differences found. Several studies have found an association of t-EAEC strains with diarrhea [6,54,55,56,57,58] and highlighted that the presence of the master regulator *aggR* is critical for EAEC pathogenesis (adherence, biofilm formation, colonization, and persistence in the intestinal gut) [32] while others not [12,18,19]. Our data reinforce the notion that EAEC is a heterogeneous pathogen causing a wide range of symptoms due to being highly diverse in virulence determinants. Other DECs pathogens (ETEC and EPEC) also detected in our surveillance and reported by Gonzales, Joffre, Rivera, Sjöling, Svennerholm and Iñiguez Gonzales, Joffre, Rivera, Sjöling, Svennerholm and Iñiguez [7] reached similar severity scores. EAEC has been associated with diarrhea as a sole pathogen or in co-infections [7]. Although our analysis of the severity of the disease excluded cases of co-infection, e.g., EAEC-rotavirus, or EAEC-ETEC, other pathogens not tested in the surveillance (i.e., norovirus, *Shigella, Campylobacter*, *Salmonella*, *Yersinia* species or parasites) might have contributed to some extent to the symptoms and masked potential true differences in the severity score between EAEC subtypes. EAEC infection has also been associated with persistent diarrhea and gut inflammation [9,10]. Boll and McCormick Boll and McCormick [59] reported that the inflammatory response might play an important role during EAEC infection. EAEC triggers inflammation through the intimate contact with the host epithelium mediated by the AAFs [9,10]. The specific association of t-EAEC isolates expressing the *aafA* gene (AAF/II) with diarrhea identified in this study might suggest higher inflammation levels during t-EAEC infections in contrast to t-EAEC or a-EAEC isolates that are lacking both AAF variants. Additional identification of the remaining AAF variants (AAF III-V) will improve the understanding of the role of AAFs in EAEC diarrhea and inflammation. The host nutritional status, such as malnourishment, has worsened EAEC infection in a murine model [60] and children [9]. A substantial proportion of the children from the Bolivian population are malnourished, with stunting on 30%, and >40% have anemia [61]. These could aggravate diarrheal onset associated with a-EAEC isolates lacking AggR. Host factors found in the children’s gut or diet might downregulate *aggR*-regulon expression and display less virulence [24]. Future studies on EAEC severity should include other pathogens of diarrheal diseases and clinical features of gut inflammation to characterize more accurately the disease caused by EAEC subtypes. Perhaps, the definition of typical and atypical based only on the presence of the *aggR* gene might introduce bias in the classification.

We further identified seven virulence genes of EAEC to better understand the EAEC strains’ heterogenicity and evaluate potential candidates as gene markers of this pathogen. We reported remarkable heterogeneity of virulence genes, of which two out of seven genes, i.e., *irp2* and *aafA*, were significantly associated with diarrhea. The high prevalence of *irp2* was comparable with previous reports [15,62,63] and highlight that siderophore production and iron scavenging is a mechanism widely used by EAEC strains. The *irp2* gene is located on a pathogenicity island, disseminated among *E. coli* species through horizontal gene transfer [64].

Adhesin AAF/II encoded by the *aafA* gene was the least prevalent adhesin and only found in 16% and 8% of EAEC isolates from diarrheal and non-diarrheal cases, respectively; however, AAF/II prevalence was higher than EAEC isolates from children in Mali [19]. AAF/II association with diarrhea was reported in another two studies [65,66]. Lima, Boisen, Silva, Havt, De Carvalho, Soares, Lima, Mota, Nataro, Guerrant and Lima Lima, Boisen, Silva, Havt, De Carvalho, Soares, Lima, Mota, Nataro, Guerrant and Lima [18] showed that combinations of other virulence factors were not included in this study, such as *aaiC*, *agg3/4C*, *agg4A*, and *aar* (previously known as *orf61)*, were associated with diarrhea compared to control samples. Both genes *irp2* and *aafA* were more prevalent among our t-EAEC than a-EAEC isolates, but they scored similar severity scores suggesting that these genes alone or in a combination of the other five investigated virulence genes might (or do) not contribute to more severe diarrhea.

The *aap* genes that encode for dispersin was the most prevalent virulence gene among all EAEC isolates; similar to our research, other studies found *aap* in high proportions [19,31,67]. Even though dispersin was also found among other diarrheagenic *E. coli* pathotypes and nonpathogenic *E. coli* [68], several studies focused on EAEC isolates have shown that *aap* gene was a highly prevalent virulent marker and associated with diarrheal cases [13,56,57,58]. Since the *aap* gene showed to be a non-exclusive EAEC virulence gene, its addition to the initial detection for EAEC will not improve the identification of EAEC isolates. Dispersin is a protein that promotes EAEC dispersion to colonize new regions of the intestine [25]. Dispersin secretion depended on the EAEC ABC transporter encoded by five genes (designated-*aatPABCD*) [25] which includes the *aatA* genes used for EAEC identification. That could explain the high frequency of *aap* gene among *aatA*-positive EAEC isolates in our study.

Here, t-EAEC harbored significantly more virulence genes than a-EAEC. Although t-EAEC harbored more virulence genes that could lead to higher virulence, t-EAEC was equally severe as a-EAEC. Several studies have also reported more genes among t-EAEC than a-EAEC isolates, but this can be because more attention has been made to characterize t-EAEC for often being associated with diarrhea [10].

The lack of *aggR* gene in half of our EAEC isolates coincide with Lima´s findings, and the authors suggested that this gene is not a useful virulence marker for diagnosis in opposite to what was suggested by other studies and that this gene alone is not a sensitive target and much less specific than the *aatA* gene [2,18]. In recent years case-control and epidemiological studies have used several EAEC virulence genes, and *aatA*, *aap*, *aagR*, *astA*, and *aafA* were among the most frequently used for EAEC diagnosis [12,13,20]. Additionally, Jenkins, Chart, Willshaw, Cheasty and Tompkins Jenkins, Chart, Willshaw, Cheasty and Tompkins [49] suggested that *aaiA*, a chromosomal -encoded gene, would improve EAEC diagnosis.

AggR is a master regulator that controls many plasmid-encoded (AAFs, AggR itself, Aap, and Aat) and chromosomal-encoded (*pheU* pathogenicity island) virulence determinants [24]. The identification of AggR-regulated genes among t-EAEC strains is expected. Certain factors are co-inherited because they are coordinately regulated by AggR and/or act in concert on a common pathogenic strategy [21]. *A* study found reduced mortality of infected *G. mellonella* larvae with *aggR* mutant strain and high virulence of atypical EAEC strains suggest that EAEC virulence appears to be related to the AggR regulon [69], but not exclusively as was observed here.

Among the EAEC toxins, *pet* was the most prevalent among diarrheal and non-diarrheal isolates, followed by the *astA,* and to a lesser extent *pic*. Pet and Pic belong to class I and II of SPATEs extracellular proteases secreted via Type V secretion system, respectively. EAST1 encoded by *astA* has similarities to STa ETEC enterotoxin; both can increase the secretion of chloride, particularly EAST1 has been associated with secretory diarrhea [70,71]. These toxins were not found in all EAEC isolated from diarrheal cases, and they were also present in non-diarrheal EAEC isolates, suggesting that potentially other toxins, e.g., SHET1 encoded by *set* gene, or *sepA* (secreted autotransporter toxin) found associated with diarrheal disease [19], or the combination of multiple factors/toxins are responsible for diarrhea.

The high percentage of MDR found among clinical isolates of EAEC is of great concern and reflects an extensive misuse or overuse of antimicrobials in younger Bolivian children in opposition to WHO recommendations. The use of antibiotics early in life may influence the immune system’s structure and functions and cause profound alteration in the microbiota, leading to impaired growth and increased susceptibility to infectious diseases [72]. Our findings correlate with studies in Bolivia and other developing countries where widespread use of antibiotics can be purchased over the counter and without any prescription [73]. Studies in La Paz showed that diarrheagenic *E. coli* are 60–90% resistant to the most common antibiotics, such as ampicillin, tetracycline trimethoprim-sulfamethoxazole [7,74,75]. Among healthy Bolivian children, 63% carried MDR-*E. coli* in their stool samples [76]. Similar results were found in EAEC from children in Kenya with persistent diarrhea [77] as well as in the aEPEC isolates from the GEMS study (carried out at seven sites in South Asia and sub-Saharan Africa) [78]. In particular, we observed that trimethoprim-sulfamethoxazole had been broadly used to treat diarrhea since a significant number of EAEC isolates were resistant to this antibiotic in comparison with non-diarrheal isolates. To our knowledge, there have been no reports on the antibiotic susceptibility on atypical EAEC. According to our results, ampicillin and trimethoprim-sulfamethoxazole should not be considered to be used to treat bacterial diarrhea, whereas ciprofloxacin, due to low levels of resistance among EAEC strains, can be only recommended in severe cases of EAEC infection with or without malnourishment, to decrease mortality for acute diarrhea. More recent AMR monitoring among EAEC isolates must be done before considering the antibiotic of choice for treating severely affected patients since AMR in bacterial isolates is already high and continues on the rise [73].

In this study, it was financially challenging to include additional virulence genes in the molecular diagnosis approach for EAEC identification as it is done and recommended by several other studies [10,19,31]. A more sensitive pathogen detection could improve our ability to study EAEC diversity and association with diarrhea. Finally, a possible methodological limitation of this study is that children from the non-diarrheal group were older than those with diarrhea. As EAEC is endemic in Bolivia and causes infection mainly among children younger than two years of age, children with non-diarrheal cases probably were previously exposed or infected by EAEC, resulting in the acquisition of immunity [79] or long-term carriers. Additional studies on the virulence of these strains isolated from non-diarrheal patients need to be done to determine whether they are truly pathogenic or not.

The result of this work is to bring attention to local and regional stakeholders and the global health community about the importance of EAEC as a common diarrheal pathogen and the alarming levels of MDR bacteria associated with infant diarrhea. Despite our limitations in the characterization of the diversity EAEC, we believe that our data together with the identification of the seasonal patterns and the levels of multidrug resistance among clinical isolates could help to encourage effective interventions, preventions, and awareness that could lead to better diagnosis and treatment and the mitigation of burden of MDR in bacteria.

In conclusion, our study is the first study in Bolivia, focusing on an integral characterization of the diarrheal diseases caused by EAEC and bacterial isolates and has uncovered knowledge and understanding of the epidemiology of EAEC and its heterogeneity in the region. We showed that EAEC is a circulating endemic pathogen responsible for a large proportion of hospitalization cases for acute diarrhea in children, particularly during the transition of seasons. A large proportion of bacterial strains displayed multidrug resistance to the most frequently used antibiotics. Here we found that, contrary to what is suggested in the literature, the EAEC subtype a-EAEC lacking the transcriptional factor AggR, was responsible for most cases of diarrhea considering that AggR is a global virulence regulator and its presence correlated with the presence of the pAA and indicates a vast virulence arsenal. Diarrhea caused by this subtype was as severe as t-EAEC, even though the last harbored more virulence genes reported to play an important role in the diarrheal disease. To clarify the pathogenicity of a-EAEC and t-EAEC, further epidemiological and extensive genomics studies are needed.

## 4. Methods

### 4.1. Ethical Permits

The present study was conducted as part of the Diarrheal Disease Project and the National Rotavirus Surveillance program in Bolivia. The Bolivian National Bioethics Committee granted both programs’ ethical permits to collect stool samples from children less than five years of age hospitalized with or without acute diarrhea to identify bacterial and viral diarrheagenic pathogens [7]. Upon arrival at the hospital, the parents or guardians of the children were informed about the purpose of the study, asked to participate and provide their oral consent to collect and analyze the fecal samples from the participants, including the clinical information of the hospitalization symptoms. The sample collection was non-invasive and directly taken after deposition or from the diapers. Participant data and/or specimens were anonymous and stored without the collection of any identification that may link to a specific individual. Oral informed consent was requested instead of the written form since parents or guardians of the participants may be illiterate due to the lack of formal education. The same procedure for all the participants was carried out. All information was given either in Spanish or their native language, i.e., Aymara or Quechua.

### 4.2. Fecal Samples

From January 2007 to December 2010, a total of 3943 stool samples of hospitalized children less than five years with acute diarrhea were collected as part of the National Diarrhea Surveillance (NDS), which included five hospitals in two cities in Bolivia (La Paz: Hospital Materno Infantil, Boliviano Holandés, Hospital del Niño, and Hospital Los Andes, and Cochabamba: Albina Patiño). In parallel, stool samples of 1026 children less than five years, with no diarrhea record, were collected [7]. The NDS evaluated the main diarrheagenic *E. coli* (DEC) categories, such as enteroaggregative *E. coli* (EAEC), enterotoxigenic *E. coli* (ETEC), enteropathogenic *E. coli* (EPEC), enterohaemorrhagic *E. coli* (EHEC), and enteroinvasive *E. coli* (EIEC). Stool samples where EAEC was isolated as sole DEC pathogen were included in this study for further PCR screening of virulence genes and antibiotic susceptibility tests.

### 4.3. Clinical Manifestations, the Severity of the Disease, and Weather Information

Data of patients enrolled in the study for characterization of the population study, e.g., age, sex, and date of hospitalization, as well as the features of acute diarrhea for the evaluation of the severity of the disease, were collected from the patient’s clinical records obtained from the different participating hospitals. A modified version of the 20-points scores Vesikari score that integrates the gastrointestinal symptoms of the diarrhea disease into scores was used to evaluate the severity of diarrhea (as described by Gonzales, Joffre, Rivera, Sjöling, Svennerholm and Iñiguez [7]). The modified Vesikari score included duration of diarrhea (1 to 3 points), the maximum frequency of stools per day (0 to 3), the maximum frequency of vomits per day (0 to 3), dehydration (0 to 4), treatment by oral or intravenous rehydration (1 to 3), presence of metabolic acidosis (0 to 2), and electrolytic disequilibrium (0 to 2). The patient’s total of points was classified as follows: mild severity from 0 to 8 points; moderate from 9 to 14 and severe from 15 to 20. The top score is 20.

For analysis of the seasonal distribution of EAEC infection, data concerning temperature and precipitation were extracted from the National Service of Hydrology and Meteorology of Bolivia (SENAHM) (http://senamhi.gob.bo/index.php/inicio).

### 4.4. Bacterial Isolation and EAEC Identification

Cary-Blair tubes containing feces swaps were transported from the hospitals to the laboratories at the Instituto de Biología Molecular y Biotecnología (IBMB) in La Paz be cultured on *E. coli* Medium (EC) and streaked onto MacConkey agar for isolation of lactose-positive fermenting bacterial colonies. As was described elsewhere [7], five lactose-positive fermenting colonies per sample were collected, the DNA extracted by boiling (15 min at 99 °C), centrifuged at 16,000× *g* for 3 min, and the supernatants pooled into one tube. 10 µL of supernatant was used as a DNA template for gene-targeted PCR assay using the gene *aatA* as a virulence marker for EAEC identification (Table S4). Each reaction was performed in a 20 µL reaction volume that contained 10 µL of the template DNA, 0.5 nM dNTPs (Promega), 1.5 mM or two mM MgCl_2_ (for multiplex PCR), 2 U GoTaq^®^ DNA polymerase (Promega), and 10 pmol of each primer. Samples *aatA*-positive were stored at −70 °C in Trypticase soy broth (TSB) containing 15% glycerol for further PCR characterization.

### 4.5. Detection of Virulence Factors by PCR

For identifying EAEC virulence genes, the bacterial DNA from each isolate was extracted as was described above, and the supernatant used as a template for targeting seven virulence genes using two panels of multiplex-PCR assays and one single PCR (Table S4). PCR reaction preparation was done as described above. PCR conditions for one single PCR and I-EAEC multiplex were as follow: one cycle for 5 min at 95 °C; 35 cycles for 30 s at 95 °C, 30 s at 57 °C and 1 min at 72 °C; 10 min at 72 °C. For II-EAEC multiplex: one cycle for 5 min at 95 °C; 30 cycles for 30 s at 95 °C, 45 s at 55 °C and 1.5 min at 72 °C; 10 min at 72 °C. The resulting PCR products were electrophoresed onto gels with 2.5% Agarose, and the DNA bands were visualized and photographed under UV light after staining with ethidium bromide. EAEC strain 042 and 17–2 have been used as a positive control [80], and nonpathogenic *E. coli* K12 (ATCC 47076) has been used as a negative control for the PCR assay.

### 4.6. Antibiotic Susceptibility Testing

The determination of the sensibility to antibiotics was done using the disk diffusion method according to the Clinical and Laboratory Standards Institute (CLSI) guidelines (Wayne 2011). The following antibiotic disks were used: ampicillin (25 g), ampicillin-sulbactam (20 g), cefotaxime (30 g), cefoxitin (30 g), ciprofloxacin (5 g), chloramphenicol (30 g), nalidixic acid (30 g), tetracycline (30 g) and trimethoprim-sulfamethoxazole (1:19) (Oxoid^®^). The *Escherichia coli* ATCC^®^ 25,922 and *Staphylococcus aureus* ATCC^®^ 25,923 were used as reference strains. Strains that exhibit resistance to one or more agents in the last three different antimicrobial categories are considered multidrug-resistant (MDR) [81].

### 4.7. Statistical Analysis and Data Visualization

The program GraphPad Prism version 8.4.2 was used to evaluate the statistical significance of the differences between the groups by the χ2-test or Fisher’s exact test (whenever necessary). All *p* values reported are two-sided. *p* < 0.05 was considered to be statistically significant. The virulence gene profile and the antimicrobial resistance test data were used to generate cluster analysis and evaluate relatedness between EAEC isolates. For data conversion, score one was given for the presence of a gene or phenotypic resistance to a specific antibiotic, and the score 0 was given when the isolate lacks the virulence gene or the isolate was considered susceptible. The euclidian distance was used for clustering, and the heatmaps were generated using the R package *pheatmap v1.012*. Bar plots for time series plots of EAEC seasonal distribution were generated using the R package *ggplot2*.

## Figures and Tables

**Table 1:** Prevalence of enteroaggregative *Escherichia coli* (EAEC) and EAEC subtypes among diarrheal and non-diarrheal cases from Bolivian children less than five years of age included in the study. t-EAEC: typical EAEC, a-EAEC: atypical EAEC.

EAEC Subtype	Diarrheal Cases		Non-Diarrheal Cases		Total	OD Ratio (95% CI)	*p*-Value
	(*n* = 3943)		(*n* = 1026)				
	n	(%)	n	(%)			
EAEC	440	(11.2)	74	(7.2)	514	1.62 (1.25–2.09)	0.0001
a-EAEC	254	(6.4)	37	(3.6)	291	1.84 (1.31–2.61)	0.0004
t-EAEC	186	(4.7)	37	(3.6)	223	1.32 (0.92–1.90)	0.1497

**Table 2:** Clinal symptoms and severity scores of pediatric subjects with diarrhea caused by t-EAEC or a-EAEC among Bolivian children. Numbers are mean +/- SD or n (%).

Variable	Typical EAEC		Atypical EAEC		Total EAEC	
	(*n =* 81)		(*n =* 78)		(*n =* 159)	
Mean Maximum Frequency of Stools Per Day	6.95 ± 3.3		7.36 ± 3.7		7.14 ± 3.4	
Mean maximum frequency of vomits per day	5.17 ± 3.1		4.92 ± 3.6		4.79 ± 3.5	
Mean no. of days with diarrhea	3.48 ± 2.4		3.92 ± 3.1		3.95 ± 4.1	
Dehydration:						
None	8	(9.9)	6	(7.7)	14	(8.8)
Mild	50	(61.7)	54	(69.2)	104	(65.4)
Severe	23	(28.4)	18	(23.1)	41	(25.8)
Treatment:						
Oral	17	(21.0)	10	(12.8)	27	(17.0)
Intravenous	64	(79.0)	66	(84.6)	130	(81.8)
Complications:						
Electrolytic disequilibrium	13	(16.0)	22	(28.2)	35	(22.0)
Metabolic acidosis	21	(25.9)	24	(30.8)	45	(28.3)
Vesikari Score:						
Mild (0–8)	17	(21.0)	19	(24.4)	36	(22.6)
Moderate (9–14)	59	(72.8)	53	(67.9)	112	(70.4)
Severe (15–20)	5	(6.2)	6	(7.7)	11	(6.9)
Mean Vesikari score	10.9 ± 3.0		10.76 ± 2.7		10.85 ± 2.9	

**Table 3:** Prevalence of virulence genes of EAEC isolated from diarrheal and non-diarrheal cases among Bolivian children. ns = nonsignificant.

Virulence Genes	Diarrheal Isolates (D)				Total (*n =* 440)		*p*-Value (D)	Non-Diarrheal Isolates (ND)				Total (*n =* 74)		*p*-Value (ND)	*p*-Value (D vs. ND)
	t-EAEC (*n =* 186)		a-EAEC (*n =* 254)					t-EAEC (*n =* 37)		a-EAEC (*n =* 37)					
	*n*	%	*n*	%	*n*	%		*n*	%	*n*	%	*n*	%		
*aap*	163	(87.6)	191	(75.2)	354	(80.5)	0.002	32	(86.5)	23	(62.2)	55	(74.3)	0.03	ns
*irp2*	155	(83.3)	193	(76.0)	348	(79.1)	ns	18	(48.6)	23	(62.2)	41	(55.4)	ns	0.001
*pic*	109	(58.6)	132	(52.0)	241	(54.8)	ns	22	(59.5)	20	(54.1)	42	(56.8)	ns	ns
*astA*	92	(49.5)	104	(40.9)	196	(44.5)	ns	20	(54.1)	11	(29.7)	31	(41.9)	ns	ns
*aggA*	38	(20.4)	47	(18.5)	85	(19.3)	ns	7	(18.9)	4	(10.8)	11	(14.9)	ns	ns
*aafA*	40	(21.5)	33	(13.0)	73	(16.6)	0.020	5	(13.5)	1	(2.7)	6	(8.1)	ns	0.039
*pet*	36	(19.4)	32	(12.6)	68	(15.5)	ns	7	(18.9)	2	(5.4)	9	(12.2)	ns	ns

**Table 4:** Prevalence of antimicrobial resistance (AMR) phenotypes of EAEC isolated from diarrheal and non-diarrheal cases among Bolivian children. ns = nonsignificant.

Drug Class	Antibiotics	Diarrheal Isolates (D)							Non-Diarrheal Isolates (ND)							*p*-Value (D vs. ND)
		t-EAEC (172)		a-EAEC (239)		Total (*n =* 411)		*p*-Value (D)	t-EAEC (30)		a-EAEC (27)		Total (*n =* 57)		*p*-Value (ND)	
		*n*	(%)	*n*	(%)	*n*	(%)		*n*	(%)	*n*	(%)	*n*	(%)		
**Penicillins**	**AM**	158	(91.9)	226	(94.6)	384	(93.4)	ns	28	(93.3)	21	(77.8)	49	(86.0)	ns	ns
	**SAM**	88	(51.2)	128	(53.6)	216	(52.6)	ns	18	(60.0)	13	(48.1)	31	(54.4)	ns	ns
**Cephalosporins**	**FOX**	24	(14.0)	20	(8.4)	44	(10.7)	ns	6	(20.0)	6	(22.2)	12	(21.1)	ns	0.0468
	**CTX**	12	(7.0)	6	(2.5)	18	(4.4)	0.0447	3	(10.0)	4	(14.8)	7	(12.3)	ns	0.0023
**Chloramphenicol**	**CHL**	38	(22.1)	54	(22.6)	92	(22.4)	ns	5	(16.7)	5	(18.5)	10	(17.5)	ns	ns
**Fluoroquinolones**	**CIP**	1	(0.6)	4	(1.7)	5	(1.2)	ns	2	(6.7)	1	(3.7)	3	(5.3)	ns	ns
	**NAL**	30	(17.4)	49	(20.5)	79	(19.2)	ns	9	(30.0)	6	(22.2)	15	(26.3)	ns	ns
**Tetracyclines**	**TET**	100	(58.1)	115	(48.1)	215	(52.3)	ns	19	(63.3)	12	(44.4)	31	(54.4)	ns	ns
**Sulphonamides**	**SXT**	117	(68.0)	189	(79.1)	306	(74.5)	ns	21	(70.0)	10	(37.0)	31	(54.4)	ns	0.0024
	**MDR**	96	(55.8)	153	(64.0)	249	(60.6)	ns	19	(63.3)	11	(40.7)	30	(52.6)	ns	ns

## Supplementary Materials

The following are available online at https://www.mdpi.com/1422-0067/21/24/9543/s1.
