# Supplementary material for: Molecular Epidemiology of Enteroaggregative Escherichia coli (EAEC) Isolates of Hospitalized Children from Bolivia Reveal High Heterogeneity and Multidrug-Resistance
Source: Int J Mol Sci. 2020 Dec 15;21(24):9543. doi: 10.3390/ijms21249543 (PMC7765457; doi:10.3390/ijms21249543)
Supplement: Supplementary file 1 [file ijms-21-09543-s001.pdf]

Supplementary Material

Table S1. Distribution of cases of EAEC and subtypes based on age groups among children less than 5 years of age with and without diarrhea.

EAEC (no. of isolates)	Age groups (month)	Diarrheal cases		Non-diarrheal cases		OD ratio (95% CI)	*P*-value
		n	(%)	n	(%)		
t-EAEC (186)	< 6	36	(19.3)	6	(16.2)	1.01 (0.40 - 2.34)	>0.9999
	7-12	76	(40.9)	16	(43.3)	0.75 (0.41 - 1.38)	0.4119
	>12	74	(39.8)	15	(40.5)	0.79 (0.42 - 1.5)	0.5063
a-EAEC (254)	< 6	40	(15.8)	3	(8.1)	2.36 (0.78 - 7.47)	0.1774
	7-12	108	(42.5)	10	(27.0)	2.08 (1.05 - 4.26)	0.0369
	>12	106	(41.7)	24	(64.9)	0.66 (0.39 - 1.12)	0.1488
all EAEC (440)	< 6	76	(17.3)	9	(12.2)	1.48 (0.73 - 3.08)	0.3947
	7-12	184	(41.8)	26	(35.1)	1.33 (0.78 - 2.23)	0.3080
	>12	180	(40.9)	39	(52.7)	0.62 (0.38 - 1.00)	0.0748

Table S2. Distribution of EAEC cases according to the season and age groups. D = diarrheal cases; ND = non-diarrheal cases.

Pathogen	Variable	Warm-humid*		Cold-dry		OD ratio (95% CI)	*p*-value
		n	(%)	n	(%)		
EAEC	D (440)	216	(49.1	224	(50.9)	0.6575 (0.3926 - 1.095)	0.1039
	ND (74)	44	(59.5)	30	(40.5)		
	a-EAEC	130	(51.2)	124	(48.8)	1.219 (0.8398 - 1.776)	0.3346
	t-EAEC	86	(46.2)	100	(53.8)		
	**Age groups (months)**						
a-EAEC (254)	< 6	19	(7.5)	21	(8.3)	0.9323 (0.4958 - 1.830)	0.8695
	7-12	53	(20.9)	55	(21.7)	0.9991 (0.6484 - 1.557)	>0.9999
	>12	58	(22.8)	48	1(8.9)	1.346 (0.8596 - 2.076)	0.2199
t-EAEC (186)	< 6	12	(6.5)	24	(12.9)	0.4902 (0.2452 to 0.9791)	0.056
	7-12	39	(21.0)	37	(19.9)	1.114 (0.6887 to 1.809)	0.7061
	>12	35	(18.8)	39	(21.0)	0.9173 (0.5566 to 1.497)	0.7991

Table S3. Prevalence of virulence genes combinations among EAEC isolates

Genotypes	Diarrheal isolates (n=440)		Non-diarrheal isolates (n=74)		Total	
	**n**	**%**	**n**	**%**	**n**	**%**
aap irp pic	32	7.3	2	2.7	34	6.6
aap irp pic aggR	27	6.1	3	4.1	30	5.8
aap irp pic astA	25	5.7	3	4.1	28	5.4
aap irp	18	4.1	5	6.8	23	4.5
aap irp aggR	20	4.5	1	1.4	21	4.1
aap irp pic astA aggR	17	3.9	2	2.7	19	3.7
aap	15	3.4	3	4.1	18	3.5
irp	13	3.0	4	5.4	17	3.3
aap irp pic astA aggR aafA pet	15	3.4	1	1.4	16	3.1
aap aggR	8	1.8	5	6.8	13	2.5
aap irp pic astA aggA	11	2.5	1	1.4	12	2.3
aap irp astA aggR aggA	9	2.0	2	2.7	11	2.1
aap irp astA	10	2.3	1	1.4	11	2.1

aap irp pic astA aafA pet	9	2.0	1	1.4	10	1.9
aap irp pic astA aggR aafA	9	2.0	1	1.4	10	1.9
aap irp pic astA aggR aggA	10	2.3	0	0.0	10	1.9
pic	3	0.7	6	8.1	9	1.7
aap irp astA aggA	7	1.6	2	2.7	9	1.7
aap irp pic astA aggR pet	6	1.4	2	2.7	8	1.6
aap pic	6	1.4	2	2.7	8	1.6
aap irp pic astA pet	7	1.6	1	1.4	8	1.6
aap irp pic aggA	8	1.8	0	0.0	8	1.6
aap irp astA aggR	5	1.1	2	2.7	7	1.4
irp pic	5	1.1	2	2.7	7	1.4
aap irp aggA	7	1.6	0	0.0	7	1.4
aap irp pic pet	7	1.6	0	0.0	7	1.4
irp pic astA	5	1.1	1	1.4	6	1.2
aap pic aggR	3	0.7	2	2.7	5	1.0
aap pic astA	4	0.9	1	1.4	5	1.0
aap pic astA aggR	4	0.9	1	1.4	5	1.0
irp pic aggR	4	0.9	1	1.4	5	1.0
aap irp aafA	5	1.1	0	0.0	5	1.0
aap irp aggR aggA	5	1.1	0	0.0	5	1.0

irp astA	5	1.1	0	0.0	5	1.0
aap irp pic aggR aggA	4	0.9	0	0.0	4	0.8
aap irp pic aggR pet	4	0.9	0	0.0	4	0.8
irp aggR	4	0.9	0	0.0	4	0.8
aap astA aggR	1	0.2	2	2.7	3	0.6
aap pic aggR aggA	1	0.2	2	2.7	3	0.6
aap aggR aggA	3	0.7	0	0.0	3	0.6
aap astA	3	0.7	0	0.0	3	0.6
aap irp pic astA aafA	3	0.7	0	0.0	3	0.6
irp astA aggA	3	0.7	0	0.0	3	0.6
irp pic astA aggR	3	0.7	0	0.0	3	0.6
irp pic astA aggR aafA	3	0.7	0	0.0	3	0.6
irp pic astA aggR aafA pet	3	0.7	0	0.0	3	0.6
aap aggA	1	0.2	1	1.4	2	0.4
irp astA aggR	1	0.2	1	1.4	2	0.4
pic aggR	1	0.2	1	1.4	2	0.4
aap aafA	2	0.5	0	0.0	2	0.4
aap astA aafA pet	2	0.5	0	0.0	2	0.4
aap irp aggR aafA	2	0.5	0	0.0	2	0.4
aap irp astA aggR aafA pet	2	0.5	0	0.0	2	0.4
aap irp pic aggR aafA pet	2	0.5	0	0.0	2	0.4

aap irp pic astA aafA aggA pet	2	0.5	0	0.0	2	0.4
aap pic aggA	2	0.5	0	0.0	2	0.4
astA aggA	2	0.5	0	0.0	2	0.4
irp astA aafA	2	0.5	0	0.0	2	0.4
irp pet	2	0.5	0	0.0	2	0.4
irp pic astA aggR aggA	2	0.5	0	0.0	2	0.4
aap pic astA aggR	0	0.0	1	1.4	1	0.2
aap irp astA aggR aafA	0	0.0	1	1.4	1	0.2
aap irp aggR pet	0	0.0	1	1.4	1	0.2
aap irp pic astA aggR aafA aggA	0	0.0	1	1.4	1	0.2
aap irp pic astA aggR aafA aggA pet	0	0.0	1	1.4	1	0.2
aap irp pic astA aggR aggA pet	0	0.0	1	1.4	1	0.2
irp pic astA aggR pet	0	0.0	1	1.4	1	0.2
pic irp aggR	0	0.0	1	1.4	1	0.2
aafA	1	0.2	0	0.0	1	0.2
aap aggR aafA pet	1	0.2	0	0.0	1	0.2
aap astA aafA	1	0.2	0	0.0	1	0.2
aap astA aggA	1	0.2	0	0.0	1	0.2
aap irp astA aggR	1	0.2	0	0.0	1	0.2
aap irp astA aggR aafA aggA pet	1	0.2	0	0.0	1	0.2

aap irp aafA aggA	1	0.2	0	0.0	1	0.2
aap irp aggR aafA pet	1	0.2	0	0.0	1	0.2
aap irp astA aggR aafA aggA	1	0.2	0	0.0	1	0.2
aap irp pic aafA	1	0.2	0	0.0	1	0.2
aap irp pic aafA pet	1	0.2	0	0.0	1	0.2
aap pic aafA	1	0.2	0	0.0	1	0.2
aap pic aggR aggA pet	1	0.2	0	0.0	1	0.2
aggR	1	0.2	0	0.0	1	0.2
astA	1	0.2	0	0.0	1	0.2
irp aafA	1	0.2	0	0.0	1	0.2
irp astA aggR aggA	1	0.2	0	0.0	1	0.2
irp pic aggA	1	0.2	0	0.0	1	0.2
irp pic astA aafA	1	0.2	0	0.0	1	0.2
irp pic astA pet	1	0.2	0	0.0	1	0.2
irp pic pet	1	0.2	0	0.0	1	0.2
pic aggA	1	0.2	0	0.0	1	0.2
Negative	14	3.2	1	1.4	15	3.1

Table S4. Primers used in this study.

Multiplex	Target Gene	Primer Sequence (5′to 3′)	Amplicon Size (bp)	Ref.
	aatA (pCVD432)	F: CTGGCGAAAGACTGTATCAT<br>R:CAATGTATAGAAATCCGCTGTT	630	[1]
	aggR	F: CGT TCG TGA ATG CAA AG<br>R: GAG GCA TCA CCA ATA TG	308	[2]
I-EAEC	*astA*	F:CCATCAACACAGTATATCCGA<br>R:GGTCGCGAGTGACGGCTTTGT	111	[1]
	pic	F:TTCAGCGGAAAGACGAA<br>R:TCTGCGCATTCATACCA	517	[3]
	irp2	F:AAGGTTCGCTGTTACCGGAC<br>R:TCGTCGGGCAGCGTTTCTTCT	267	[4]
II-EAEC	*aggA*	F:GCTAACGCTGCGTTAGAAAGACC<br>R:GGAGTATCATTCTATATTCGCC	421	[3]
	aafA	F:GACAACCGCAACGCTGCGCTG<br>R:GATAGCCGGTGTAATTGAGCC	233	[3]
	pet	F:TCATTTCCAGCACTTCCTGT<br>R:CTCCGACAGTATTTGCTCGT	442	[5]
	aap	F:CTTTTCTGGCATCTTGGGT<br>R:GTAACAACCCCTTTGGAAGT	232	[1]

References

1. Kahali, S.; Sarkar, B.; Rajendran, K.; Khanam, J.; Yamasaki, S.; Nandy, R.K.; Bhattacharya, S.K.; Ramamurthy, T. Virulence characteristics and molecular epidemiology of enteroaggregative Escherichia coli isolates from hospitalized diarrheal patients in Kolkata, India. *J. Clin. Microbiol.* **2004**, *42*, 4111-4120, doi:10.1128/JCM.42.9.4111-4120.2004.
2. Nataro, J.P.; Yikang, D.; Yingkang, D.; Walker, K. AggR, a transcriptional activator of aggregative adherence fimbria I expression in enteroaggregative Escherichia coli. *J. Bacteriol.* **1994**, *176*, 4691-4699, doi:10.1128/jb.176.15.4691-4699.1994.
3. Piva, I.C.; Pereira, A.L.; Ferraz, L.R.; Silva, R.S.N.; Vieira, A.C.; Blanco, J.E.; Blanco, M.; Blanco, J.; Giugliano, L.G. Virulence markers of enteroaggregative Escherichia coli isolated from children and adults with diarrhea in Brasília, Brazil. *J. Clin. Microbiol.* **2003**, *41*, 1827-1832, doi:10.1128/jcm.41.5.1827-1832.2003.
4. Schubert, S.; Rakin, A.; Karch, H.; Carniel, E.; Heesemann, J. Prevalence of the "high-pathogenicity island" of Yersinia species among Escherichia coli strains that are pathogenic to humans. *Infection and immunity* **1998**, *66*, 480-485, doi:10.1128/IAI.66.2.480-485.1998.
5. Yatsuyanagi, J.; Saito, S.; Sato, H.; Miyajima, Y.; Amano, K.; Enomoto, K. Characterization of enteropathogenic and enteroaggregative Escherichia coli isolated from diarrheal outbreaks. *J Clin Microbiol* **2002**, *40*, 294-297, doi:10.1128/jcm.40.1.294-297.2002.
